# Ankle-brachial index in HIV infection

**DOI:** 10.1186/1742-6405-6-6

**Published:** 2009-04-27

**Authors:** Julián Olalla, Daniel Salas, Javier de la Torre, Alfonso del Arco, José Luis Prada, Francisco Martos, Emilio Perea-Milla, Javier García-Alegría

**Affiliations:** 1 Unidad de Medicina Interna, Hospital Costa del Sol, Marbella, Spain; 2 Facultad de Medicina, Departamento de Farmacología, Universidad de Málaga, Málaga, Spain; 3 Unidad de Investigación, Hospital Costa del Sol, Marbella, Spain; 4 CIBER Epidemiología y Salud Pública (CIBERESP), Spain

## Abstract

Prognosis for patients with the human immunodeficiency virus (HIV) has improved with the introduction of highly active antiretroviral therapy (HAART). Evidence over recent years suggests that the incidence of cardiovascular disease is increasing in HIV patients. The ankle-brachial index (ABI) is a cheap and easy test that has been validated in the general population. Abnormal ABI values are associated with increased cardiovascular mortality. To date, six series of ABI values in persons with HIV have been published, but none was a prospective study. No agreement exists concerning the risk factors for an abnormal ABI, though its prevalence is clearly higher in these patients than in the general population. Whether this higher prevalence of an abnormal ABI is associated with a higher incidence of vascular events remains to be determined.

## Introduction

The generalised use of highly active antiretroviral therapy (HAART) in patients with the human immunodeficiency virus (HIV) has led to a spectacular increase in their survival rates [1-3]. At the same time, several cohort studies have noted an increase in the relative risk of major cardiovascular events, especially acute myocardial infarction and subclinical vascular disease [4-8]. Pathophysiological evidence exists that both HIV and HAART can affect the lipid profile [9,10], insulin resistance [11,12] and the vascular response to vasodilatation [13]. This has led to greater interest among physicians attending these patients in earlier diagnosis and treatment of the traditional cardiovascular risk factors, and recommendations have even been made for proactive changes in HAART, with a view to improving the profile of these risk factors [14].

Cohort studies have revealed an increase in the relative risk of acute myocardial infarction and cardiovascular disease, which has not yet been reflected in a large number of cases because absolute levels of incidence remain low [15,16]. Nevertheless, the incidence observed is greater than that expected according to predictive techniques such as Framingham's equation [17]. Given the possibility that in a few years cardiovascular disease among HIV patients will become more significant than at present, it is of interest to make use of diagnostic tests that enable the early identification of groups of patients presenting high vascular risk, so that a closer control may be maintained of cardiovascular disease risk factors. Cardiovascular disease is the fourth cause of death among HIV patients, close behind non-AIDS-associated neoplasias [18]. Ideally, such tests should be cheap, comparable, innocuous and applicable at outpatient clinics. Techniques for the early diagnosis of atherosclerotic diseases in these patients would enable persons at high risk of cardiovascular events to receive more aggressive therapy for the management of these risk factors and even enable proactive changes in their HAART to be made.

In this context, it is of interest to examine techniques such as measuring the carotid artery intima-media thickness (IMT) or the ankle-brachial index (ABI). The latter index reflects the relation between systolic arterial tension measured in the upper and in the lower limbs; values lower than 0.9 or higher than 1.3, according to guidelines to clinical practice [19], are considered pathologic and associated with a higher incidence of vascular morbimortality. A recent meta-analysis of the general population, using the ABI index, found an association of values of ≤ 1.1 or ≥ 1.4 with an increased risk of cardiovascular disease and death [20]. This meta-analysis considered 16 studies with a total of 480,325 patients-year, and concluded that an ABI ≤0.9 was associated with a doubled risk of 10-year global mortality, of cardiovascular mortality and of the incidence of severe coronary events, in comparison with the predictions derived from Framingham's equation. In fact, the application of the ABI test to patients previously stratified for vascular risk by Framingham's equation resulted in the reclassification of almost 19% of male patients and of 36% of female patients, and the consequent modification of treatment recommendations for these patients. As remarked above, current American clinical practice guidelines for peripheral arterial disease consider an abnormal ABI to be <0.9 or ≥1.3. The technique is cheap, harmless and reproducible, with a predictable inter-examiner variation [21]. In this short review, we analyze the series published on the application of ABI tests among HIV-infected populations.

## Analysis of Published Series

To date, six series have been published on patients with HIV infection and for whom the ABI was calculated. As shown in Table 1, the series are heterogeneous for both the type of patients (sex and age) and for the ABI measurement. The selection of patients was also very heterogeneous; Periard et al [22] selected patients referred from outpatient clinics, aged over 40 years, after having excluded active drug addicts and those patients with previous arterial complications; Palacios et al [23] selected patients aged over 50 years, and Bernal et al [24], those with two or more traditional cardiovascular risk factors; Sharma et al [25] included only women patients, while Gutiérrez et al [26] and Olalla et al [27] did not exclude any type of patient and did not report any conditions for inclusion. In all cases, however, the patients had been referred from HIV infection clinics. Only two of the series reported the number of patients with a high ABI [25,27], whereas all the series recorded the prevalence of an ABI <0.9. Table 2 shows the different rates of prevalence of the traditional cardiovascular risk factors in the various cohorts.

**Table 1:** Prevalence of abnormal ABI in each series

Study	Type of patients	N	Male (%)	Age in years (mean)	ABI≤0.9 N (%)	ABI≥1.3 N (%)	ABI≥1.4 N (%)
Sharma et al. [25]	Women (73.9% black)	238	0	39.6	3 (0.9)^a^	NM	17 (7.2)
Periard et al. [22]	Age >40 years	92	76.1	49.5	19 (20.7)^b^	NM	NM
Bernal et al. [24]	With ≥2 CVRF	91	87.9	50	4 (4.39)	NM	NM
Gutiérrez et al. [26]	Consecutive patients	139	72.7	45.8	4 (2.88)	NM	4 (2.88)
Palacios et al. [23]	Age ≥50 years	99	82.8	58.6	10 (10.2)	NM	NM
Olalla et al. [27]	Consecutive patients	147	82.3	43.9	4 (2.7)	29 (19.7)	8 (5.4)^c^

^a^: the prevalence of 0.9% refers to the joint cohort of women with and without HIV (total of 335 patients). No report is given of the separate prevalence for each group.

NM: not mentioned.

^b^: the prevalence includes ABI <0.9 at rest and after exercise (9.8% and 10.9%, respectively).

CVRF: cardiovascular risk factors

NM: not mentioned.

^c^: data not published; the report only refers to the total number of patients with ABI <0.9 or >1.3.

**Table 2:** Prevalence of traditional vascular risk factors in each series.

Risk factor (prevalence in %)	Sharma *et al *[25]	Periard *et al *[22]	Bernal *et al *[24]	Gutiérrez *et al *[26]	Palacios *et al *[23]	Olalla *et al *[27]
Diabetes	9.7	4.3	17.6	10.1	31.3	2
Hypertension	23.4	27.2	57.1	28.8	36.4	4.1
Dyslipidaemia	NM	NM	69.2	NM	69.4	18.4
Hypertriglyceridemia	13.4	35.9	NM	NM	NM	NM
High LDL cholesterol	6	17.4	NM	NM	NM	NM
Low HDL cholesterol	26.2	13	NM	NM	NM	NM
Cigarette smoking	43.5	62	72.5	61.1	30.3	59.9
Family history of cardiovascular events	NM	22.8	19.8	10.8	15.2	2.7

NM: not mentioned.

Both Sharma and Palacios [23,25] compared the prevalence of altered ABI in patients with and without HIV infection, although among very different types of patients. Sharma et al [25] compared HIV-infected women aged, on average, 39.6 years with non-infected women aged, on average, 36.4 years (p = 0.002) and found the infected women to include a higher proportion of patients with HDL cholesterol ≤35 mg/dl (26.2% vs 5.2%, p < 0.001) and of triglycerides ≥200 mg/dl (13.4% vs 5.2%, p = 0.03). However, despite the difference in age and the poorer metabolic profile of HIV-infected patients, the prevalence of increased ABI was similar (7.2% among HIV infected patients vs 6.3% among non-infected patients). The prevalence of low ABI was only 0.9% (among the two groups of patients). The series analyzed by Palacios et al [23] was constituted basically of male patients (82.8%), with the HIV-group presenting a higher proportion of smokers (30.3% vs 46.5%, p = 0.02), and a higher body mass index (24.8 kg/m^2 ^vs 27.7 kg/m^2^, p = 0.0001), while the HIV+ group presented a higher proportion of patients with hyperlipemia (69.4% vs 36.7%, p = 0.0001), diabetes (31.3% vs 12.2%, p = 0.002) and cardiovascular risk >20% calculated by Framingham's equation (29.5% vs 13.4%, p = 0.008). The prevalence of ABI <0.9 was significantly greater among the HIV+ patients than among those not so infected (10.2% vs 1%, p = 0.01).

All the studies were cut-off studies, and no follow-up study has yet been made. Only Gutiérrez et al [26] studied the association between an abnormal ABI and a marker of cardiovascular disease, the carotid artery intima-media thickness (IMT). They found that an ABI <0.9 was associated with an increased IMT, though the same was not found for patients with an ABI >1.4. Except Sharma et al [25], the studies have been conducted mainly in men.

As regards factors associated with an abnormal ABI, Sharma et al. undertook a multivariate analysis of a group of women with and without HIV. They established that cigarette smoking (OR: 2.53; 95% CI, 0.99–6.43), a body mass index <18.5 (OR: 11; 95% CI, 1.61–75.63) and overweight (OR 5.4; 95% CI, 1.13–25.89) were all associated with an increased ABI. Periard et al [22] found that factors predicting a low ABI were age (OR 1.09; 95% CI, 1–1.18, for each additional year), cigarette smoking (OR 1.7; 95% CI, 1.17–2.46, for each additional 10 pack-years), diabetes (with a perfect prediction, as all the diabetic patients included in the multivariate analysis had a low ABI) and a CD4 cell count below 200 cells per microlitre (OR 27.2; 95% CI, 2.55–286.01). Bernal et al [24] and Palacios et al [23] found no significant association with a low ABI. A univariate analysis by Gutiérrez et al [26] of factors related with a low ABI found significant differences between the number of classical cardiovascular factors (4 in patients with a low ABI versus 2 in the others, p = 0.015) and a lower CD4 cell count (220 vs. 450 cells per microlitre, p = 0.009). In their multivariate analysis, Olalla et al [27] related it with the use of protease inhibitors (OR 2.79; 95% CI, 1.15–6.54) and the presence of dyslipidemia (OR 2.68; 95% CI, 1.06–6.91); this study, too, found a significant difference in CD4 cells in patients with an abnormal ABI (185.64 vs. 266.67 cells/mL, p = 0.03).

## Discussion

The prevalence of an abnormal ABI in patients with HIV is greater than in the general population, especially regarding those with a high ABI. It remains to be determined whether the current cut-off points for the non-infected population (which leave out the population at risk, such as those with an ABI between 0.9 and 1.1) have the same value in the population with HIV. These cut-off points need to be validated with follow-up studies on the incidence of major ischemic cardiovascular events. Analysis of the different series clearly shows that the prevalence of an abnormal ABI is far more frequent in persons with HIV compared with the general population, in whom the prevalence of peripheral arterial disease is estimated to be 1% at the age of 50 years and 3% at the age of 60 [28,29].

Of note in our patients was the high prevalence of an ABI >1.3 or >1.4. Whilst the prevalence of an ABI <0.9 is higher in HIV infected persons than among the general population, a high ABI is even more prevalent. A meta-analysis recently published by the Ankle Brachial Index Collaboration [20] showed that an ABI ≥1.4 was associated with greater overall mortality and cardiovascular mortality in both men and women; this association was not found for an ABI ≥1.3. The same deleterious association was also established for an ABI ≤ 1.1. Clinical practice guidelines, however, still retain the cut-off points of 0.9 and 1.3 when referring to a pathological ABI [19].

The prevalence of altered ABI varies greatly among the different series analyzed. At one extreme is that of Periard [22], who reported a prevalence of ABI <0.9 of 20.7%. The selection criterion used in this case was merely that of patients aged over 40 years, even though the average age was in fact close to 50 years. The reason for this high rate of prevalence may lie in the fact that 15% of the patients in this series reported intermittent claudication, according to the Edinburgh questionnaire, which inclines us to believe that selection bias may be present. At the other extreme is the series described by Sharma [25], in which the prevalence of ABI <0.9 was only 0.9%; this value corresponded to a group of women with an average age of less than 40 years.

Studies such as those by Periard [22], Gutiérrez [26] and Olalla [27] suggest a possible association between a higher degree of immunosuppression and altered ABI. The first of these authors assigned an OR of 27 to obtain an altered ABI if CD4 < 200 cells/microlitre, while Gutiérrez and Olalla found a significant difference in the CD4 lymphocyte cell count between altered and non-altered ABI. Studies have been made of other subordinate markers, such as the carotid intima-media thickness; these, too, relate one-year progression with higher levels of immunosuppression. HIV itself has revealed an inverse relation between the level of viral load and endothelium-mediated vasodilation, and also with components of the antiretroviral treatment applied, such as protease inhibitors or abacavir [13,30]. This would account for findings such as those for Olalla's series [27], in which protease inhibitors were associated with altered ABI, this effect being controlled by the presence of dyslipidaemia. Periard [22] reported an OR of 1.03, with no statistically significant association, with the accumulated use of protease inhibitors.

Smoking has been associated with both a low ABI and with a high ABI, probably because, on the one hand, it is involved in the generation and progression of atheromatous plaques, while on the other; it affects the elasticity of the arterial wall.

This same toxicity towards the endothelium, expressed as the induction of apoptosis (ritonavir) or as the stimulation of endothelium-mediated vasodilation, may partially account for the relation between the use of protease inhibitors with altered ABI, especially due to the lack of arterial compliance (ABI >1.3) rather than because of pure stenosis of the arterial lumen (ABI <0.9). Thus, the high prevalence of a high ABI may be mediated by the involvement of vascular elasticity as well as by the generation of atheroma plaques.

All the series published show a prevalence of altered ABI that is much greater than that of the rates of peripheral arterial disease observed among the general population: 1% at the age of 50 years and 3% at 60 years. If large series confirm this finding, a greater and earlier incidence of vascular events would be expected among the HIV-infected population. Indeed, various studies have already reported a greater and earlier incidence of cardiovascular disease among these patients [31,32].

Framingham's equation underestimates the vascular risk affecting HIV-infected patients [17]. If ABI measuring becomes generalised, those patients with pathological values should be considered to be at high cardiovascular risk and hence management of cardiovascular risk factors should be more aggressive than usual.

## Conclusion

Vascular risk has become an important issue in HIV infected people. Prevalence of abnormal ABI appears to be higher in these patients, and PI use could be in relationship with this. Generalized use of ABI could be an interesting way to identify patients with high vascular risk.

## Competing interests

The authors declare that they have no competing interests.

## Authors' contributions

Conception and design: JO, JT, AA, DS. Revision of the different versions of the study protocol: JO, EPM. Collection and assembly of data: DS. Quality control of the data: FM, JO, EPM. Analysis and interpretation of the data: JO, DS, FM. Drafting of the article: JO. Critical revision of the article for important intellectual contents: JGA. Final approval of the article: JO, JT, AA, JLP.

## References

[B1] Lewden C, Chene G, Morlat P, Raffi F, Dupon M, Dellamonica P, Pellegrin JL, Katlama C, Dabis F, Leport C (2007). HIV-infected adults with a CD4 cell count greater than 500 cells/mm3 on long-term combination antiretroviral therapy reach same mortality rates as the general population. J Acquir Immune Defic Syndr.

[B2] Lima VD, Hogg RS, Harrigan PR, Moore D, Yip B, Wood E, Montaner JS (2007). Continued improvement in survival among HIV-infected individuals with newer forms of highly active antiretroviral therapy. AIDS.

[B3] Palella FJ, Delaney KM, Moorman AC, Loveless MO, Fuhrer J, Satten GA, Aschman DJ, Holmberg SD (1998). Declining morbidity and mortality among patients with advanced human immunodeficiency virus infection. HIV Outpatient Study Investigators. N Engl J Med.

[B4] Friis-Moller N, Sabin CA, Weber R, d'Arminio MA, El Sadr WM, Reiss P, Thiebaut R, Morfeldt L, De Wit S, Pradier C, Calvo G, Law MG, Kirk O, Phillips AN, Lundgren JD (2003). Combination antiretroviral therapy and the risk of myocardial infarction. N Engl J Med.

[B5] Friis-Moller N, Weber R, Reiss P, Thiebaut R, Kirk O, d'Arminio MA, Pradier C, Morfeldt L, Mateu S, Law M, El Sadr W, De Wit S, Sabin CA, Phillips AN, Lundgren JD (2003). Cardiovascular disease risk factors in HIV patients – association with antiretroviral therapy. Results from the DAD study. AIDS.

[B6] Friis-Moller N, Reiss P, Sabin CA, Weber R, Monforte A, El Sadr W, Thiebaut R, De Wit S, Kirk O, Fontas E, Law MG, Phillips A, Lundgren JD (2007). Class of antiretroviral drugs and the risk of myocardial infarction. N Engl J Med.

[B7] Hsue PY, Lo JC, Franklin A, Bolger AF, Martin JN, Deeks SG, Waters DD (2004). Progression of atherosclerosis as assessed by carotid intima-media thickness in patients with HIV infection. Circulation.

[B8] Johnsen S, Dolan SE, Fitch KV, Kanter JR, Hemphill LC, Connelly JM, Lees RS, Lee H, Grinspoon S (2006). Carotid intimal medial thickness in human immunodeficiency virus-infected women: effects of protease inhibitor use, cardiac risk factors, and the metabolic syndrome. J Clin Endocrinol Metab.

[B9] Bernal E, Masia M, Padilla S, Gutierrez F (2008). High-density lipoprotein cholesterol in HIV-infected patients: evidence for an association with HIV-1 viral load, antiretroviral therapy status, and regimen composition. AIDS Patient Care STDS.

[B10] Kannel WB, Giordano M (2004). Long-term cardiovascular risk with protease inhibitors and management of the dyslipidemia. Am J Cardiol.

[B11] Mulligan K, Grunfeld C, Tai VW, Algren H, Pang M, Chernoff DN, Lo JC, Schambelan M (2000). Hyperlipidemia and insulin resistance are induced by protease inhibitors independent of changes in body composition in patients with HIV infection. J Acquir Immune Defic Syndr.

[B12] Murata H, Hruz PW, Mueckler M (2000). The mechanism of insulin resistance caused by HIV protease inhibitor therapy. J Biol Chem.

[B13] Hsue P, Wu Y, Schnell A, Ganz P, Hunt P, Hatano H, Martin J, Deeks S (2009). Association of Abacavir and HIV Disease Factors with Endothelial Function in Patients on Long-term Suppressive ART. 16th Conference on Retroviruses and Opportunistic Infections, Montreal 2009 Abstract 723.

[B14] Lundgren JD, Battegay M, Behrens G, De Wit S, Guaraldi G, Katlama C, Martinez E, Nair D, Powderly WG, Reiss P, Sutinen J, Vigano A (2008). European AIDS Clinical Society (EACS) guidelines on the prevention and management of metabolic diseases in HIV. HIV Med.

[B15] Lundgren J, Reiss P, Worm SW, Weber R, El Sadr W, De Wit S, Law M, d'Arminio, Monforte A, Pradier C, Sabin CA (2009). Risk of Myocardial Infarction with Exposure to Specific ARV from the PI, NNRTI, and NRTI Drug Classes: The D:A:D Study. 16th Conference on Retroviruses and Opportunistic Infections, Montreal 2009 Abstract 44LB.

[B16] Sabin CA, Worm SW, Weber R, Reiss P, El Sadr W, Dabis F, De Wit S, Law M, d'Arminio, Monforte A, Friis-Moller N, Kirk O, Pradier C, Weller I, Phillips AN, Lundgren JD (2008). Use of nucleoside reverse transcriptase inhibitors and risk of myocardial infarction in HIV-infected patients enrolled in the D:A:D study: a multi-cohort collaboration. Lancet.

[B17] Law MG, Friis-Moller N, El Sadr WM, Weber R, Reiss P, d'Arminio MA, Thiebaut R, Morfeldt L, De Wit S, Pradier C, Calvo G, Kirk O, Sabin CA, Phillips AN, Lundgren JD (2006). The use of the Framingham equation to predict myocardial infarctions in HIV-infected patients: comparison with observed events in the D:A:D Study. HIV Med.

[B18] Weber R, Sabin CA, Friis-Moller N, Reiss P, El Sadr WM, Kirk O, Dabis F, Law MG, Pradier C, De Wit S, Akerlund B, Calvo G, Monforte A, Rickenbach M, Ledergerber B, Phillips AN, Lundgren JD (2006). Liver-related deaths in persons infected with the human immunodeficiency virus: the D:A:D study. Arch Intern Med.

[B19] Hirsch AT, Haskal ZJ, Hertzer NR, Bakal CW, Creager MA, Halperin JL, Hiratzka LF, Murphy WR, Olin JW, Puschett JB, Rosenfield KA, Sacks D, Stanley JC, Taylor LM, White CJ, White J, White RA, Antman EM, Smith SC, Adams CD, Anderson JL, Faxon DP, Fuster V, Gibbons RJ, Hunt SA, Jacobs AK, Nishimura R, Ornato JP, Page RL, Riegel B (2006). ACC/AHA 2005 Practice Guidelines for the management of patients with peripheral arterial disease (lower extremity, renal, mesenteric, and abdominal aortic): a collaborative report from the American Association for Vascular Surgery/Society for Vascular Surgery, Society for Cardiovascular Angiography and Interventions, Society for Vascular Medicine and Biology, Society of Interventional Radiology, and the ACC/AHA Task Force on Practice Guidelines (Writing Committee to Develop Guidelines for the Management of Patients With Peripheral Arterial Disease): endorsed by the American Association of Cardiovascular and Pulmonary Rehabilitation; National Heart, Lung, and Blood Institute; Society for Vascular Nursing; TransAtlantic Inter-Society Consensus; and Vascular Disease Foundation. Circulation.

[B20] Fowkes FG, Murray GD, Butcher I, Heald CL, Lee RJ, Chambless LE, Folsom AR, Hirsch AT, Dramaix M, deBacker G, Wautrecht JC, Kornitzer M, Newman AB, Cushman M, Sutton-Tyrrell K, Fowkes FG, Lee AJ, Price JF, d'Agostino RB, Murabito JM, Norman PE, Jamrozik K, Curb JD, Masaki KH, Rodriguez BL, Dekker JM, Bouter LM, Heine RJ, Nijpels G, Stehouwer CD, col (2008). Ankle brachial index combined with Framingham Risk Score to predict cardiovascular events and mortality: a meta-analysis. JAMA.

[B21] Baker JD, Dix DE (1981). Variability of Doppler ankle pressures with arterial occlusive disease: an evaluation of ankle index and brachial-ankle pressure gradient. Surgery.

[B22] Periard D, Cavassini M, Taffe P, Chevalley M, Senn L, Chapuis-Taillard C, de Valliere S, Hayoz D, Tarr PE (2008). High prevalence of peripheral arterial disease in HIV-infected persons. Clin Infect Dis.

[B23] Palacios R, Alonso I, Hidalgo A, Aguilar I, Sanchez MA, Valdivielso P, Gonzalez-Santos P, Santos J (2008). Peripheral Arterial Disease in HIV Patients Older than 50 Years of Age. AIDS Res Hum Retroviruses.

[B24] Bernal E, Masia M, Padilla S, Hernandez I, Gutierrez F (2008). Low prevalence of peripheral arterial disease in HIV-infected patients with multiple cardiovascular risk factors. J Acquir Immune Defic Syndr.

[B25] Sharma A, Holman S, Pitts R, Minkoff HL, Dehovitz JA, Lazar J (2007). Peripheral arterial disease in HIV-infected and uninfected women. HIV Med.

[B26] Gutierrez F, Bernal E, Padilla S, Hernandez I, Masia M (2008). Relationship between ankle-brachial index and carotid intima-media thickness in HIV-infected patients. AIDS.

[B27] Olalla J, Salas D, Del Arco A, De la TJ, Prada J, Machin-Hamalainen S, Garcia-Alegria J (2009). Ankle-branch index and HIV: the role of antiretrovirals. HIV Med.

[B28] Murabito JM, Evans JC, Nieto K, Larson MG, Levy D, Wilson PW (2002). Prevalence and clinical correlates of peripheral arterial disease in the Framingham Offspring Study. Am Heart J.

[B29] Zheng ZJ, Rosamond WD, Chambless LE, Nieto FJ, Barnes RW, Hutchinson RG, Tyroler HA, Heiss G (2005). Lower extremity arterial disease assessed by ankle-brachial index in a middle-aged population of African Americans and whites: the Atherosclerosis Risk in Communities (ARIC) Study. Am J Prev Med.

[B30] Francisci D, Giannini S, Baldelli F, Leone M, Belfiori B, Guglielmini G, Malincarne L, Gresele P (2009). HIV type 1 infection, and not short-term HAART, induces endothelial dysfunction. AIDS.

[B31] Grinspoon SK, Grunfeld C, Kotler DP, Currier JS, Lundgren JD, Dube MP, Lipshultz SE, Hsue PY, Squires K, Schambelan M, Wilson PW, Yarasheski KE, Hadigan CM, Stein JH, Eckel RH (2008). State of the science conference: Initiative to decrease cardiovascular risk and increase quality of care for patients living with HIV/AIDS: executive summary. Circulation.

[B32] Triant VA, Lee H, Hadigan C, Grinspoon SK (2007). Increased acute myocardial infarction rates and cardiovascular risk factors among patients with human immunodeficiency virus disease. J Clin Endocrinol Metab.

